# Molecular Mechanisms of Vitamin D-Mediated Immunomodulation

**DOI:** 10.31661/gmj.v10i0.2097

**Published:** 2021-06-05

**Authors:** Farhan Khashim Alswailmi, Syed Imran Ali Shah, Haleema Nawaz, Ghassab Mohammad Al-Mazaideh

**Affiliations:** ^1^University of Hafr Al-Batin, Hafar Al Batin, Saudi Arabia; ^2^Central Park Medical College, Lahore, Pakistan

**Keywords:** Vitamin D, Immunomodulation, Innate Immunity, Adaptive Immunity

## Abstract

Ever since discovering the fat-soluble secosteroid vitamin D, an abundance of research has been conducted on the molecular mechanisms for the multiple health benefits of this nutrient. Studies on the beneficial effects of vitamin D supplementation have found appreciable evidence suggesting that it may play a more prime role than initially presumed. Though it has largely been implicated in bone pathophysiology, novel research on vitamin D indicates its fundamental involvement in a wide range of disease processes through its multiple systemic effects, including but not limited to metabolic, cardiovascular, anti-inflammatory, antineoplastic, antioxidant, neuroprotective, and immune actions. Recent work has yielded important mechanistic insights into the functions of vitamin D in mediating immunity. The present work sheds light on the metabolism and immune response mechanisms of vitamin D. Current review is based on a thorough search of the available relevant research findings of the metabolic transformations of vitamin D and the molecular basis of its role in immunity. Apart from its classical mechanistic control of mineral homeostasis, vitamin D has immunomodulatory effects through various mechanisms at both systemic and cellular levels. Disruption of vitamin D reliant molecular pathways in the regulation of immune response can potentially result in the development and/or progression of autoimmune and infective processes.

## Vitamin D Resources and Metabolism

**Figure 1:**
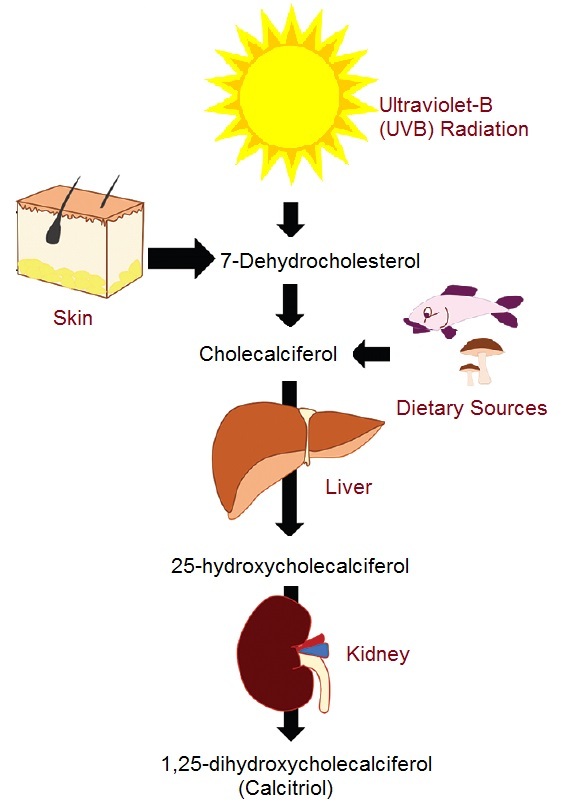
Endogenous and exogenous sources of vitamin D and its metabolic activation.

Unlike other vitamins, which can be obtained from an abundance of natural sources, vitamin D (calciferol), except fatty fish (mackerel, sardines, salmon), egg yolk, and some mushrooms, is not usually present in most foodstuff unless it has been artificially fortified. Skin exposure to ultraviolet B radiation is viewed as the chief source of vitamin D in mammals (Figure-1); however, little to no sun exposure is assumed while considering supplementation.The role of vitamin D in body function mirrors that of a hormone, with the active form calcitriol exerting its action through nuclear receptors present in numerous body tissues [1,2].Vitamin D is biologically inactive whether obtained from the diet (vitamin D2 or ergocalciferol) or synthesized within the body (vitamin D3 or cholecalciferol).Activation of the pro-hormones, ergocalciferol, and cholecalciferol, necessitates the addition of two hydroxyl groups, the first one in the liver and the second one in the kidneys (Figure-1) [3].In the liver, calciferol is converted to 25-hydroxyvitamin D (25OHD) mediated by the enzyme 25-hydroxylase (cytochrome P450 2R1[CYP2R1]).Subsequently, the renal 1α-hydroxylase (cytochrome P450 27B1 [CYP27B1]) converts 25OHD to 1,25-dihydroxyvitamin D (1,25[OH2] D), also known as calcitriol. The preponderant chemical version of vitamin D in blood is 25OHD which establishes the basis of serum testing [4]. Vitamin D binding protein is responsible for the plasma transport of vitamin D; it transports 25OHD, as well as the calciferol and calcitriol forms of vitamin D [5]. 25OHD and calcitriol are inactivated by the enzyme vitamin D3 24-hydroxylase (cytochrome p450 24A1[CYP24A1]) and form calciferol and calcitriol, respectively [3].

## Vitamin D Molecular Signaling and Classical Functions

**Figure 2:**
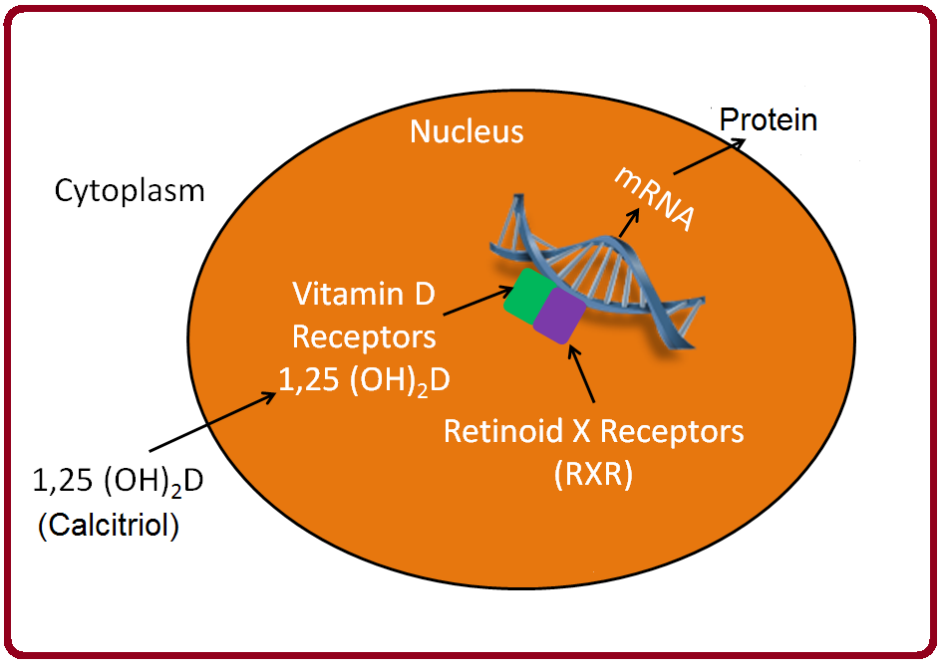
Vitamin D mediated activation of VDR and gene transcription.

Vitamin D receptor (VDR) is expressed both in the nucleus (VDRn) and on cellular membranes (VDRm) [6]. In humans, the VDR gene encoding is located on chromosome 12q [7]. VDR is widely distributed in many body tissues; this includes intestinal tissue, pancreatic beta cells, kidney tubular epithelial cells, bronchial epithelial cells, skin epithelial cells, osteoblasts, chondrocytes, certain endocrine glands, reproductive tissue, and immune cells [2].Being a nuclear steroid, Vitamin D exerts its actions through genomic and non-genomic mechanisms. The genomic actions involve binding of activated VDR with retinoid X receptor (RXR), making a complex that then interacts with vitamin D response elements (VDREs) residing on DNA, which further engenders gene transcription and subsequently protein formation (Figure-2) [8].The non-genomic action of vitamin D comprises actuation of signaling molecules and protein kinases, resulting in second messenger generation and opening of Ca+2 and Cl-channels, leading to cross-interaction with the genome and regulation of gene expression [9]. In its calcitriol form, Vitamin D is classically responsible for the regulation of calcium-phosphate balance, osteogenesis, bone remodeling, and correct parathyroid hormone function (PTH). In the intestines, calcium absorption is increased by the binding of calcitriol to VDR within the enterocyte; subsequently, via genomic action, VDR upregulates the expression of enteric calcium transporters and calbindin, a calcium-binding protein. Additionally, it increases ATP-dependent calcium pump (PMCA) activity, allowing the enterocyte to pump out greater amounts of calcium into the bloodstream [10,11]. Classically, calcitriol paired with hypophosphatemia was thought to increase expression of intestinal sodium-inorganic phosphate (Na-Pi) cotransporter type II-b (Npt2b) via VDR transcription-dependent pathway [12]. Studies have suggested that low Pi levels may increase Npt2b expression independent of vitamin D mediated transcription. However, the genomic action of vitamin D cannot be entirely dismissed as it may affect factors involved in the post-transcriptional regulation of Npt2b expression [13], thus still rendering it a contributor to intestinal Pi absorption.In the kidneys, active calcium reabsorption is seen in the distal renal tubules. Calcitriol increases renal calcium (Ca+2) uptake by stimulating nuclear transcription factors, as described above, increasing the expression of calbindin and plasma membrane Ca+2 pump. Renal tubular transport of Pi is also dependent upon the presence of calcitriol. Calcitriol increases the expression of renal Na-Pi cotransporters, types II-a and II-c, via VDR [14]. Calcitriol also affects renal Pi transport through a liposomes mechanism of action enabled by its modulatory effects on membrane phospholipid composition [15] is another important function of vitamin D in bone homeostasis. Calcitriol regulates bone resorption by increasing the differentiation of progenitor cells into mature osteoclasts and by stimulating osteoclast-like cell formation via a mechanism involving osteoblasts [16].Additionally, through its action on calcium and phosphate absorption, calcitriol enhances bone mineralization [17]. Lastly, vitamin D forms a stringent feedback cycle with PTH. PTH increases vitamin D synthesis in the kidneys, whereas vitamin D negatively affects PTH secretion [18]. Both vitamin D and PTH then exert regulatory action on bone resorption and mineralization.

##  Immunomodulatory Role of Vitamin D

The abundant expression of VDR on numerous immunologic cells, including B- and T-cells, macrophages, and dendritic cells, allows vitamin D to exert modulatory effects on both innate and adaptive immune responses [19,20]. Studies conducted on vitamin D deficient populations better highlight the pivotal role it plays in maintaining immunologic integrity. Vitamin D deficiency has been correlated with weaker immune responses, reduced phagocytosis, and decreased intracellular killing rates in microglial cells [21]. Consequently, reducing the epidermal synthesis of vitamin D during the colder season greatly increases vulnerability to respiratory infections [22]. Although most studies have targeted respiratory infections, increasing evidence supports the affiliation of vitamin D deficiency with system infection [23].Furthermore, vitamin D deficiency in the general population has been linked to increased all-cause mortality [24]. Vitamin D, therefore, is crucial in ensuring the stability of the immune system. Regular vitamin D supplementation in pregnant women has been shown to elicit more robust neonatal immune responses and a lower risk of developing childhood asthma [25]. Over the years, studies have highlighted the potential clinical use of vitamin D as a pharmacologic agent having therapeutic and preventative benefits in managing some allergic, autoimmune, infective, and oncologic conditions [26,27,28,29].

## Innate Immune System

Specific molecules preserved within the cell membranes of certain microbes called the pathogen-associated molecular patterns (PAMPs) allow the innate immune system to discern foreign invaders from host cells and respond adequately [30]. Innate immune cells express specialized proteins called pattern recognition receptors (PRRs) that are responsible for identifying PAMPs [31]. A sub-type of PRRs called toll-like receptors plays a crucial role in the innate immune response [32]. Toll-like receptor 2/1 heterodimer (TLR2/1), upon recognition of PAMPs, through intracellular signaling pathways, increases expression of VDRs and CYP27B1 within innate immune cells [33]. This ultimately results in the increased production and binding of vitamin D to VDRs. Vitamin D then stimulates the production of anti-microbial cathelicidin (CAMP) and β-defensin 2 (DEFB4) via intracellular signaling. Unlike, CAMP which only needs occupancy of vitamin D response elements, DEFB4 entails additional habitation of nuclear factor-κB (NF-κB) for transcriptional induction. Nonetheless, vitamin D is central to the generation of these anti-bacterial peptides against infectious agents [34].Another mechanism by which vitamin D augments anti-bacterial activity involves induction of intracellular pathogen-recognition receptor (NOD2) expression in various cell types, which increases cell sensitivity to muramyl-dipeptide (NOD2 ligand) produced by certain bacteria. Intracellular NF-κB is activated by NOD2, which then enhances transcription of CAMP and DEFB4 via vitamin D signaling [35]. Although unclear how vitamin D also promotes autophagy within innate immune cells [36].Additionally, intracrine vitamin D synthesis suppresses hepcidin anti-microbial peptide (HAMP) expression [37]. HAMP is known to suppress ferroportin, a cell membrane protein that exports intracellular iron. This immobilization of intracellular iron lowers circulating iron concentrations and serves as an important host response against pathogens such as bacteria which utilize iron for growth [38]. However, in the case of bacteria such as Mycobacterium tuberculosis, Chlamydia psittaci, and Salmonella typhimurium, which evade the immune system by internalization within host cells, HAMP suppression and subsequent increase in iron export prove beneficial [33]. Thus, this effect of vitamin D on iron homeostasis harmonizes with its actions on autophagy and anti-bacterial peptide expression. Vitamin D also cooperates with other factors to further strengthen the innate immune response, such as TGF-β, which acts in concert with vitamin D to increase the expression of 5-lipo-oxygenase (5-LO) enzyme that involved in the biosynthesis of leukotrienes [39]. In addition to enhancing innate immune responses, vitamin D contributes to a feedback control mechanism that prevents the over-elaboration of inflammatory events arising from excessive immune system activation. Vitamin D does this by promoting hyporesponsiveness of Toll-like receptors to PAMPs by downregulation [40].

## Antigen Presentation

By carrying out antigen presentation, antigen-presenting cells (APC) bridge the gap of innate and adaptive immune systems. Among these APCs are dendritic cells, which process antigenic material and present it to T-cells. Dendritic cells are classified most commonly as myeloid (mDCs) dendritic cells, further divided into two subsets: mDC-1 and mDC-2, and plasmacytoid (pDCs) dendritic cells. mDCs promote T-cell function, whereas pDCs generally serve to diminish it [41,42]. Vitamin D mainly regulates dendritic cell maturation, and therefore antigen presentation to T-cells. VDR and CYP27B1 are expressed by dendritic cells; however, VDR expression in immature dendritic cells is higher compared to mature dendritic cells. In contradiction, CYP27B1 expression is greater in mature dendritic cells than immature dendritic cells [43]. As a result, vitamin D produced by mature dendritic cells, in a paracrine fashion, binds to VDRs on immature dendritic and enhances cell maturation. This inconsistent expression of VDRs and CYP27B1 between mature and immature dendritic cells prevents the inflammatory sequelae of dendritic cell hyperactivation [19,33].

## Adaptive Immune System

 Independent of its effects on innate immunity and antigen presentation, vitamin D also exerts regulatory control on adaptive immunity. The presence of VDR, as well as CYP27B1 in B- and T-lymphocytes, directly links vitamin D to adaptive immunity, with VDR expression directly proportional to T- and B-cell proliferation [44]. Apart from its suppressive actions on T-cell proliferation, vitamin D is also capable of influencing T-cell phenotype by inhibiting T helper type-1 (Th-1) cells, a subtype of the cluster of differentiation 4 positive (CD4+) cells [45].Vitamin D thus reduces tissue damage resulting from unwarranted Th-1 activity by shifting to Th-2 cellular phenotype which is involved in humoral immunity [33]. Recently, vitamin D has been shown to suppress the development of Th-17 cells that produce the pro-inflammatory cytokine interleukin-17 (IL-17), which promotes immune response but has also been inculpated in tissue inflammation [46,47]. Thus, inhibition of IL-17 expression by vitamin D protects host tissues from excessive damage; this inhibition occurs at a post-transcriptional level [48].Vitamin D also exerts actions on regulatory T-cells (Tregs), which constrain proliferation of other CD4+ cells. Vitamin D stimulates Tregs development from naïve CD4+ T-cells, which may prove beneficial in autoimmune disease. Vitamin D achieves this by directly expressing VDR expression by CD4+ cells [49] and by indirect induction of immature dendritic cells to produce Tregs populations [50]. Vitamin D’s action on cytotoxic CD8+ cells is ambiguous despite an abundance of VDRs. These actions are likely sub-set specific. CD8αα cells, a subtype of CD8+ T cells, may be involved in suppressing inflammation of the gastrointestinal tract [51]. Knockout studies exhibit reduction of CD8αα cell expression in VDR deficient mice, owing to the fact that T-cell homing to the gut is mediated by VDR [52].B-cells are not exempt from vitamin D’s immunomodulatory actions as it suppresses the production and proliferation of immunoglobulins by them [53]. A more recent study reported vitamin D mediated inhibition of class-switched memory cell and plasma cell differentiation [54]. This particular effect highlights a prospective clinical role for vitamin D in autoimmune disorders related to B-cell dysfunction [27].

## Conclusion

In addition to summarizing the historically well-known molecular action of VDR signaling in bone and mineral balance, the current review has explored the multimodal actions of vitamin D on immunity. Our review presents vitamin D’s immune actions with a background on its classical effects on bone metabolism and calcium-phosphate balance, having VDR signaling at the core. We highlight the immunosuppressive potential of vitamin D and provide key mechanistic insights into a more modulatory role of vitamin D in immune homeostasis. Through its involvement at various levels of the immune system, vitamin D seems to restore immune balance in conditions associated with immune dysregulation. VDR expression in activated lymphocytes and the subsequent action of the bioactive metabolite calcitriol in response to specific antigens is indicative of the immunomodulatory potential of vitamin D. Keeping in view the above discussion, vitamin D seems to exert potent immunomodulatory effects on both innate and adaptive immune systems through several mechanisms in addition to its classical regulatory control of the skeletal and mineral balance. While adequacy of the circulating levels of vitamin D helps mediate overall immunity, VDR expression on immune cells such as B and T lymphocytes and their synthesis of active vitamin D also allows for local regulation of immune response. Vitamin D insufficiency may disrupt the molecular pathways regulating immunity and lead to autoimmune disorders and increased risk of infections and/or their progression. Further work on the molecular signaling of vitamin D in immune regulation can bring to the fore its potential ameliorative effects on immune diseases. Moreover, vitamin D’s direct causal effect on immunity can be elucidated through adequately-powered, well-designed placebo-controlled clinical supplementation studies.

## Acknowledgment

The authors thank the Deanship of Scientific Research, the University of Hafr Al-Batin, for funding the work through project No. G-103-2020.

## Conflict of Interest

None of the authors has any conflict of interest to declare for this work.
